# Blood Flow Restriction Resistance Training in Tendon Rehabilitation: A Scoping Review on Intervention Parameters, Physiological Effects, and Outcomes

**DOI:** 10.3389/fspor.2022.879860

**Published:** 2022-04-25

**Authors:** Ian Burton, Aisling McCormack

**Affiliations:** ^1^Musculoskeletal (MSK) Service, Fraserburgh Physiotherapy Department, Fraserburgh Hospital, National Health Service (NHS) Grampian, Aberdeen, United Kingdom; ^2^Aberdeen City Council, Aberdeen, United Kingdom

**Keywords:** blood flow restriction, tendinopathy, resistance training, exercise, physiotherapy, tendon

## Abstract

**Objective:**

To identify current evidence on blood flow restriction training (BFRT) in tendon injuries and healthy tendons, evaluating physiological tendon effects, intervention parameters, and outcomes.

**Methods:**

This scoping review was reported in accordance with the PRISMA Extension for Scoping Reviews (PRISMA-ScR). Databases searched included MEDLINE, CINAHL, AMED, EMBase, SPORTDiscus, Cochrane library (Controlled trials, Systematic reviews), and five trial registries. Two independent reviewers screened studies at title/abstract and full text. Following screening, data was extracted and charted, and presented as figures and tables alongside a narrative synthesis. Any study design conducted on adults, investigating the effects of BFRT on healthy tendons or tendon pathology were included. Data were extracted on physiological tendon effects, intervention parameters and outcomes with BFRT.

**Results:**

Thirteen studies were included, three on tendinopathy, two on tendon ruptures, and eight on healthy Achilles, patellar, and supraspinatus tendons. A variety of outcomes were assessed, including pain, function, strength, and tendon morphological and mechanical properties, particularly changes in tendon thickness. BFRT intervention parameters were heterogeneously prescribed.

**Conclusion:**

Despite a dearth of studies to date on the effects of BFRT on healthy tendons and in tendon pathologies, preliminary evidence for beneficial effects of BFRT on tendons and clinical outcomes is encouraging. As BFRT is a relatively novel method, definitive conclusions, and recommendations on BFRT in tendon rehabilitation cannot be made at present, which should be addressed in future research, due to the potential therapeutic benefits highlighted in this review.

## Introduction

Tendinopathy is a disease entity which can cause significant pain and functional limitations for individuals and collectively places a tremendous burden on society through high healthcare costs (Hopkins et al., 2016; Dean et al., 2017). In chronic tendinopathy, tendons experience morphological changes and can present with increased tendon thickness, fibril disorganization, and neovascularization caused by repetitive tendon microtrauma (Magnusson and Kjaer, 2019; Millar et al., 2021). Tendinopathy prevalence has been shown to be higher in athletes due to frequent jumping, landing, running and change of direction movements (Zwerver et al., 2011). Collectively, tendinopathies can account for up to 30% of all musculoskeletal conditions requiring medical attention, with up to 22% of elite athletes having patellar tendinopathy at least once during their sporting careers (Lian et al., 2005; Skjong et al., 2012; Canosa-Carro et al., 2022). Complete and partial tendon ruptures are also common in both athletes and the general population with the Achilles tendon having the highest prevalence of ruptures (Nyyssonen et al., 2008). Like tendinopathy, tendon ruptures can also cause significant pain, disability and functional limitations and are associated with significant societal and healthcare costs, whether treated surgically or conservatively, with there being a lack of consensus on optimal treatment methods (Holm et al., 2015).

Resistance training has long been considered the treatment of choice in the rehabilitation of chronic tendinopathies, with both eccentric and heavy slow resistance training (HSRT) demonstrating positive clinical effects, for both improving symptoms and tendon structure (Kongsgaard et al., 2010; Beyer et al., 2015). Progressive resistance training is also considered an essential element of rehabilitation following tendon rupture to counteract muscle atrophy and stimulate tendon repair, whether treated conservatively or surgically (Christensen et al., 2020). The application of progressive tendon loads during rehabilitation is essential to not compromise tendon healing, with the precise dosage parameters of resistance training loading a critical consideration (Bohm et al., 2015). Prolonged time under tension with traditional heavy loads during the early phase of tendon rehabilitation could be counterproductive and compromise tendon healing (Loenneke et al., 2012a; Couppe et al., 2015). Blood flow restriction training (BFRT) is a method of resistance training which utilizes pneumatic cuffs or straps around a limb to partially restrict arterial blood flow, while simultaneously occluding venous outflow until the cessation of cuff pressure (Lorenz et al., 2021). BFRT also known as occlusion, hypoxic or Kaatsu training has become increasingly popular over the last decade as a method for enhancing strength gains in healthy populations such as athletes and more recently as a rehabilitation tool in those with musculoskeletal pathologies (Hughes et al., 2017; Barber-Westin and Noyes, 2019; Nitzsche et al., 2021). For example, BFRT has been found to be an efficacious method for increasing strength gains and muscle hypertrophy in rehabilitation following surgery for anterior cruciate ligament (ACL) rupture (Hughes et al., 2018; Caetano et al., 2021). The physiological benefits associated with BFRT, include beneficial adaptations to the cardiovascular, endocrine, and musculoskeletal systems with psychosocial benefits also reported such as mood and performance improvement (Karabulut et al., 2013, 2021; Neto et al., 2016; Silva et al., 2018; Bowman et al., 2019; da Silva et al., 2019; Okita et al., 2019; Freitas et al., 2021a; Miller et al., 2021).

Whilst traditional eccentric or HSRT for tendinopathy utilizes heavy training loads of up to 70% of 1 repetition maximum (1-RM), low-load BFRT (LL-BFRT) typically uses lower training intensities, and loads in the range of 20–40% of 1RM, which may be more tolerable for patients not able to tolerate high muscle-tendon training loads, while still preventing muscle atrophy and promoting hypertrophy (Centner et al., 2019a; Krzysztofik et al., 2019; Shiromaru et al., 2019; Kataoka et al., 2022). Interventional studies have found superior or similar clinical outcomes with LL-BFRT compared to conventional high-load resistance training (HL-RT) in knee rehabilitation for ACL reconstruction, patellofemoral pain, and knee osteoarthritis (Ohta et al., 2003; Bryk et al., 2016; Giles et al., 2017; Ferraz et al., 2018; Korakakis et al., 2018a; Ferlito et al., 2020; Grantham et al., 2021). BFRT has been shown to cause exercise-induced hypoalgesia through endogenous opioid and endocannabinoid mechanisms, so could therefore be a useful pain management tool in early musculoskeletal rehabilitation, particularly in the presence of an acute pain response (Korakakis et al., 2018b; Hughes and Patterson, 2019, 2020; Hughes et al., 2021). Recent evidence suggests that LL-BFRT may be a superior method for augmenting muscular adaptations in early musculoskeletal rehabilitation, which has been found to be comparably effective for inducing muscular hypertrophy and only minimally inferior for increasing muscular strength compared to HL-RT (Manini and Clark, 2009; Abe et al., 2012; Loenneke et al., 2012b; Yasuda et al., 2012; Martin-Hernandez et al., 2013; Lixandrao et al., 2018; Hughes et al., 2019a). The mechanisms of action of BFRT in muscular adaptation are thought to be related to increased inflammation and metabolic stress which can increase blood supply to muscles potentially stimulating muscle growth (Loenneke et al., 2012c; Pearson and Hussain, 2015; Rossi et al., 2018; Freitas et al., 2021a). Other speculated physiological mechanisms explaining muscle hypertrophy adaptations in response to BFRT includes activation of chemoreceptors, muscle swelling, and increased protein synthesis (Freitas et al., 2021a). Due to a paucity of research, it is unclear what effects BFRT may have on tendons, but the induced ischemic muscular milieu may facilitate morphological and mechanical tendon properties through enhanced collagen metabolism and tendon remodeling (Klein et al., 2001; Boesen et al., 2013). Despite these potential beneficial physiological mechanisms of BFRT on tendon healing, the method of training has received a dearth of attention in tendon rehabilitation, despite the clinical benefits found for other musculoskeletal conditions and the knowledge of resistance training being the most evidence-based treatment available for tendinopathies. Therefore, the objective of this scoping review is to evaluate current research on the use of BFRT for treating tendon injuries. The scoping review will be guided by addressing the following review questions on specific aspects of BFRT interventions within tendon rehabilitation: 1. What outcomes have been reported for BFRT in healthy tendons and rehabilitation for tendon injuries and which outcome measures have been used? 2. What BFRT intervention and cuff parameters have been used in published studies? 3. What physiological mechanisms explaining effects of BFRT on tendons and tendon injuries have been investigated in published studies?

## Methods

Due to the exploratory nature of the research questions a scoping review was conducted as they are recommended for mapping key concepts, evidence gaps and types of evidence within a particular field and can help guide future research and the possibility of conducting systematic reviews on the topic (Tricco et al., 2018). The scoping review is reported in accordance with the Preferred Reporting Items for Systematic reviews and Meta-analysis extension for Scoping reviews known as the PRISMA-ScR (Tricco et al., 2018). This scoping review aimed to evaluate current BFRT interventions in healthy tendons and the rehabilitation of tendon injuries for the first time in the literature. The results will allow dissemination of the parameters of research BFRT interventions to clinical practitioners through peer-reviewed journal publication, allowing increased likelihood of implementation in clinical practice. The review will also outline future research and exercise reporting needs within BFRT interventions in tendon rehabilitation.

### Eligibility Criteria

The inclusion criteria for the scoping review were guided by a modified PICO (PCoCo) as recommended for scoping reviews (Tricco et al., 2018). Studies including adults aged 18 years or older with a diagnosis of a tendon injury for any time duration were considered. Tendon injuries included both acute partial or full tendon tears or ruptures and any chronic tendon injuries diagnosed as any tendinopathy. Any tendon condition characterized by common tendinopathy symptoms, including full thickness tendon rupture were considered for inclusion. Studies including healthy participants with no history of tendon pathology were also included. Studies including participants with other concurrent injuries or medical conditions not tendon related were excluded. The concept of interest was BFRT for healthy tendons or for the treatment of any tendon related injury, including any type or format such as BFRT performed with bodyweight or external resistance. BFRT may be delivered across a range of settings by health or exercise professionals, delivered in a supervised or unsupervised manner, using any methods for training progression and monitoring. This scoping review considered both experimental and quasi-experimental study designs including randomized controlled trials and non-randomized controlled. In addition, prospective and retrospective cohort studies, case series and case reports were considered for inclusion. Unpublished studies, reviews or reports were not considered for inclusion.

### Search Strategy

The search was carried out using a uniform search strategy across all databases (Appendix 1) and it included key words from two main concepts: Blood Flow Restriction (“Kaatsu,” “Occlusion training”), and Tendon (“tendon,” “tendinopathy,” “tendon rupture”). The Boolean operators “Or” and “And” were used to link the key words from each concept and to link the concepts themselves, respectively. A 3-step search strategy was implemented in this scoping review. It incorporated the following: (1) a limited search of MEDLINE and CINAHL using initial keywords as, followed by analysis of the text words in the title/abstract and those used to describe articles to develop a full search strategy; (2) The full search strategy was adapted to each database and applied to MEDLINE, CINAHL, AMED, EMBase, SPORTDiscus, and the Cochrane library (Controlled trials, Systematic reviews). The following trial registries were also searched: ClinicalTrials.gov, ISRCTN, The Research Registry, EU-CTR (European Union Clinical Trials Registry), ANZCTR (Australia and New Zealand Clinical Trials Registry). Databases were searched from inception to March 1st, 2022 (Search performed on March 1st, 2022). The search for relevant gray literature included Open Gray, MedNar, Cochrane central register of controlled trials (CENTRAL), EThOS, CORE, and Google Scholar. (3) For each article located in steps 1 and 2, a search of cited and citing articles using Scopus and hand-searching where necessary, was conducted. Studies published in a language other than English were only considered if a translation was available as translation services are not available to the authors.

### Study Selection

Following the search, all identified citations were collated and uploaded into RefWorks and duplicates removed. Titles and abstracts were then screened by two independent reviewers for assessment against the inclusion criteria for the review. Potentially relevant studies were retrieved in full, and their citation details imported into Covidence (Veritas Health Innovation, Melbourne, Australia). Two independent reviewers assessed the full text of selected citations in detail against the inclusion criteria. Any disagreements that arose between the reviewers at each stage of the study selection process were resolved through discussion or by input from a third reviewer. The results of the search are reported in accordance with the PRISMA-ScR (Tricco et al., 2018). In accordance with guidance on conducting scoping reviews, critical appraisal was not conducted (Tricco et al., 2018).

### Data Extraction

Data were extracted from sources included in the scoping review by one reviewer, with independent data extraction by a second reviewer for at least 10% of studies. The data extracted included specific details regarding the population, concept, context, study methods and key findings relevant to the review questions. The data extracted included dimensions such as study type, purpose, population and sample size, methods, details of the BFRT intervention, specific exercises and outcome measures used. Details of the BFRT interventions included type, dosage, cuff parameters, and methods used to progress and adjust the training stimulus. Data were also be extracted on any physiological mechanisms which have been investigated to explain the effects of BFRT on tendons, and positive clinical outcomes. Decreased muscle size and strength are associated with tendon injuries, both for risk and a consequence of pathology. Therefore, data on muscle strength and size outcomes will also be extracted as improvements in muscle size and strength would be positive clinical outcomes in tendon rehabilitation, although not directly related to physiological tendon changes. The extracted data are presented in Table 1 with a narrative synthesis accompanying the tabulated results.

**Table 1:** Characterizes of included studies and BFRT intervention parameters.

**Author, Study design, population**	**Intervention, exercises, duration**	**Training parameters**	**Cuff parameters**	**Outcome measures**	**Outcomes, results**
Skovlund et al. (2020) Case series, *n* = 7, Patellar tendinopathy	Low-load BFRT: SL leg press, knee extension, 3 weeks	Sets: 6, Reps: 5–30, Freq: 3 × WK, Prog: increase volume based on pain response, Int: 10RM, (30% of 1RM). Maximum 105 reps per session	Polyester cuff (15 cm wide) fitted at proximal thigh. Occlusion pressure: 120 mm Hg Cuff pressure released for 3 Min between exercises.	Pain (NRS-P, SLDS), Function (VISA-P) Tendon vascularity (US), Knee extensor strength (MVC – static dynamometry)	Intervention was effective for improving clinical outcomes. Pain with SLDS reduced by 50%. Tendon vascularity diminished by 31% following 3 weeks. No changes in tendon thickness. Increase in knee extensor strength. Adherence: 98%
Cuddeford and Brumitt (2020) Case report, *n* = 1, Patellar tendinopathy	Low-load BFRT: SL leg press, SLDS, 12 weeks	Sets:4, Reps 15–30; Freq 2 × WK: Prog: increase resistance (10lbs Inc), Int: 15-30RM (1RM testing)	Delfi tourniquet system fitted at proximal lower extremity. Occlusion pressure: 80% restriction of arterial inflow. 30 second rest between sets (cuff not removed)	Pain (VAS), Function (VISA-P), Tendon size US, Hip and knee strength (handheld dynamometry, SL leg press 1RM)	Patients improved clinical outcomes and returned to sports activity. Improvements in tendon thickness and resolution of hypoechoic region. Increased lower limb strength Adherence: supervised.
Sata (2005), Case report, *n* = 1, Patellar tendinopathy	Low-load BFRT: straight leg raises, hip abduction and adduction, calf raise, toe raise, squat, crunch, back extension, basketball shooting, 3 weeks	Sets: 3, Reps; 15, Freq: 5-6 × WK, Prog: Int:15rm (30% of 1RM)	Kaatsu cuff fitted at proximal lower limb. Occlusion pressure range: 160–180 mmHg.	MRI (signal intensity). Thigh circumference	Patient improved clinical outcomes and returned to playing basketball. MRI signal intensity was reduced, and the thigh circumference was increased by 7 mm and 2 mm for the right and left sides. Adherence: NR
Wentzell (2018), Case report, *n* = 1, Biceps tendon rupture	Manual therapy, laser therapy, progressive strength training including Low-load BFRT: Isometric forearm pronation & supination, elbow flexion & extension 14 weeks	Sets: 4, Reps: 30,15,15,15, Freq: 7 × WK, Prog: increase resistance (1.5-4lbs) difficulty and ROM, Int: 10-30% MVC	Blood pressure cuff fitted at proximal arm. Occlusion pressure: 80 mmHg.	Pain (NPRS), Function DASH, Mayo Elbow Performance Index score.	Patient improved clinical outcomes and returned to preinjury activity (weightlifter). Adherence: NR
Yow et al. (2018) Case report, *n* = 2, Achilles tendon rupture	Low-load BFRT: Leg press, calf press, 6 weeks	Sets: 4, Reps: 30,15,15,15, Freq: NR, Prog: NR, Int: 30% of 1RM	Delfi tourniquet system (14 cm wide) fitted at proximal thigh. Occlusion pressure: 80%, 180 mm Hg.	Strength and power (isokinetic testing—Biodex system).	Patients improved strength and power and returned to sports. Adherence: NR
Centner et al. (2019b) RCT, *n* = 55, Healthy Achilles tendon	1. Low-load BFRT: standing and seated calf raises (20-35% 1RM). 2. High load RT (70-85% 1RM). 3. Non-exercise control, 14 weeks	Sets:3, Reps;6-12, Freq: 3 × WK, Prog: increase resistance (5% of 1rm every 4 WK, 20–35%), Int: 20–35% of 1RM Rest: 1 MIN between sets, 3 MIN between exercises	Pneumatic nylon tourniquet (12 cm wide) fitted on proximal thigh. Occlusion pressure: 50% arterial occlusion. Pressure maintained during 1 MIN rest; cuff deflated during 3 MIN rest.	Tendon morphology, Mechanical and material properties (US), and muscle (US) cross-sectional area (CSA) and isometric strength (MVC—isokinetic dynamometer).	Both groups induced significant increases in tendon stiffness and CSA, which were comparable between groups. Gastrocnemius CSA and plantar flexor strength significantly increased in both groups. No changes in control group. Adherence: supervised
Centner et al. (2021) RCT, *N* = 29, Healthy patellar tendon	1. Low-load BFRT: bilateral leg press and knee extension, standing and seated calf raises (20–35% 1RM) 2. High load RT (70–85% 1RM), 14 weeks	Sets: 4, Reps: 30,15,15,15, Freq: 3 × WK, Prog: increase resistance (5% of 1rm every 4 WK, 20–35%), Int: 20–35% of 1RM Rest: 1 MIN between sets, 3 MIN between exercises	Pneumatic nylon tourniquet (12 cm wide) fitted on proximal thigh. Occlusion pressure: 50% arterial occlusion. Pressure maintained during 1 MIN rest; cuff deflated during 3 MIN rest.	Tendon morphology, mechanical and material properties (US and MRI), and muscle (MRI) cross-sectional area (CSA) and strength (dynamic 1RM).	Both groups induced significant increases in tendon stiffness and CSA, muscle mass and strength, which were comparable and not significantly different between groups. Knee extension 1RM was higher in BFRT group. Adherence: supervised
Chulvi-Medrano et al. (2020) RCT, *n* = 56, Healthy Achilles tendon	1. LL BFRT: plantarflexion 2. LL RT, single session	Sets:3, Reps; 15, Freq: single session, Prog: NR, Int: 30% of 1RM Rest: 30 s between sets	High precision compression meter (57 cm long × 9 cm wide) fitted on proximal thigh. Occlusion pressure: 30%.	Tendon thickness (US)	BFRT group had significantly greater decrease in tendon thickness compared to LL-RT, immediately and 24 h after exercise, which may be associated with neurotendinous fluid movement in response to BFRT. Adherence: NR
Gavanda et al. (2020) RCT, *n* = 21, Healthy achilles tendon	1. LL BFRT: plantarflexion 2. LL RT, 6 weeks	Sets:4, Reps; to muscular failure, Freq: 2 × WK, Prog: occlusion pressure increased every 4 WKs, Int: 30% of 1RM, Rest: 30 s between sets	Twist lock (7 cm wide) cuffs fitted below patella. Occlusion pressure: 60%.	Calf volume and muscle thickness (US), maximal hopping test for leg stiffness, 1-RM smith machine calf raise, pain (VAS)	Leg stiffness and calf volume did not change. VAS, 1RM, and muscle thickness improved equally in both groups. No difference found in leg stiffness between groups: used to measure tendon adaptations. Adherence: NR
Kubo et al. (2006), Cohort, *n* = 9, Healthy patellar tendon	1. LL BFRT (20% of 1RM): plantarflexion 2. HL RT (80% of 1RM), 12 weeks	Sets:4, Reps; 25, 18, 15, 12, Freq: 3 × WK, Prog: NR, Int: 20% of 1RM Rest: 30 s between sets	Elastic pneumatic belt fitted on proximal thigh. Occlusion pressure: 37.7%.	Knee extension MVC (dynamometer) and muscle volume. Specific tension of vastus lateralis (VL), Tendon stiffness (US)	Both groups significantly increased MVC and muscle volume of quadriceps. Tension of VL increased significantly 5.5% for HL, but not for LL. Tension and tendon properties were found to remain following LL-BFRT, whereas they increased significantly after HL-RT. BFRT did not alter tendon stiffness, while the HL protocol increased it significantly. Adherence: NR
Picon-martinez et al. (2021) RCT, *n* = 52, healthy achilles tendon	1. LL BFRT (30% 1RM): plantarflexion 2. LL RT (30% 1RM) 3. HL RT (75% 1RM), single session	Sets:4, Reps; 30, 15, 15, 15, Freq: single session, Prog NR, Int: 30% of 1RM, Rest: 30 s between sets	Pneumatic CUFF (9 cm width) fitted under knee joint. Occlusion pressure: 30%.	Achilles tendon thickness (US): immediately, 60MIN and 24 h after training.	Achilles tendon thickness was significantly reduced immediately after, 60 min and 24 h post-LL BFRT group and remained unchanged in the other groups. Adherence: NR
Brumitt et al. (2020) RCT, *n* = 46, healthy supraspinatus tendon	1. LL BFRT: side-lying external rotation 2. LL RT, 8 weeks	Sets:4, Reps; 30, 15, 15, 15, Freq: 2 × WK, Prog: NR, Int: 30% of 1RM Rest: 30 s between sets	Delfi tourniquet system fitted at proximal upper arm. Occlusion pressure: 50%,	Rotator cuff strength (dynamometry), supraspinatus tendon thickness (US)	BFRT did not augment rotator cuff strength gains or tendon thickness when compared to RT. Both groups significantly increased rotator cuff strength and supraspinatus tendon thickness, with no significant difference between groups. Adherence: supervised
Canfer et al. (2021) cross sectional, *n* = 12, healthy achilles tendon	1. LL BFRT: bodyweight SL heel raise 2. LL RT	Sets:4, Reps; 30, 15, 15, 15, Freq: single session, Prog: NR, Int: 30% of 1RM Rest: 30 s between sets	Occlusion cuff (7 cm) fitted at distal lower leg. Occlusion pressure: 80%. Cuff only deflated after 4th set.	Thermograms to assess Achilles tendon skin temperature (Tskin)	A lower Tskin was seen following BFRT exercise at the tendon insertion, but not at the free tendon or the musculotendinous junction. A significant effect of time upon changes in Tskin were observed in both groups. Adherence: NR

*LL-BFRT, low-load blood flow restriction training; HL-RT, high load resistance training; RM, repetition maximum; Tskin, skin temperature; SL, single leg; US, ultrasound; MRI, magnetic resonance imaging; MIN, minute; NR, not reported; Int, intensity; Freq, frequency; Prog, Progression; RCT, randomized controlled trial; VL, vastus lateralis; MVC, maximum voluntary contraction; VAS, visual anologue scale; NRS-P, pain numeric rating scale; VISA-P, Victorian Institute of Sport Assessment Patellar; SLDS, single leg decline squat; n, number; WK, week; ROM, range of motion*.

## Results

### Included Study Characteristics

**Figure 1:**
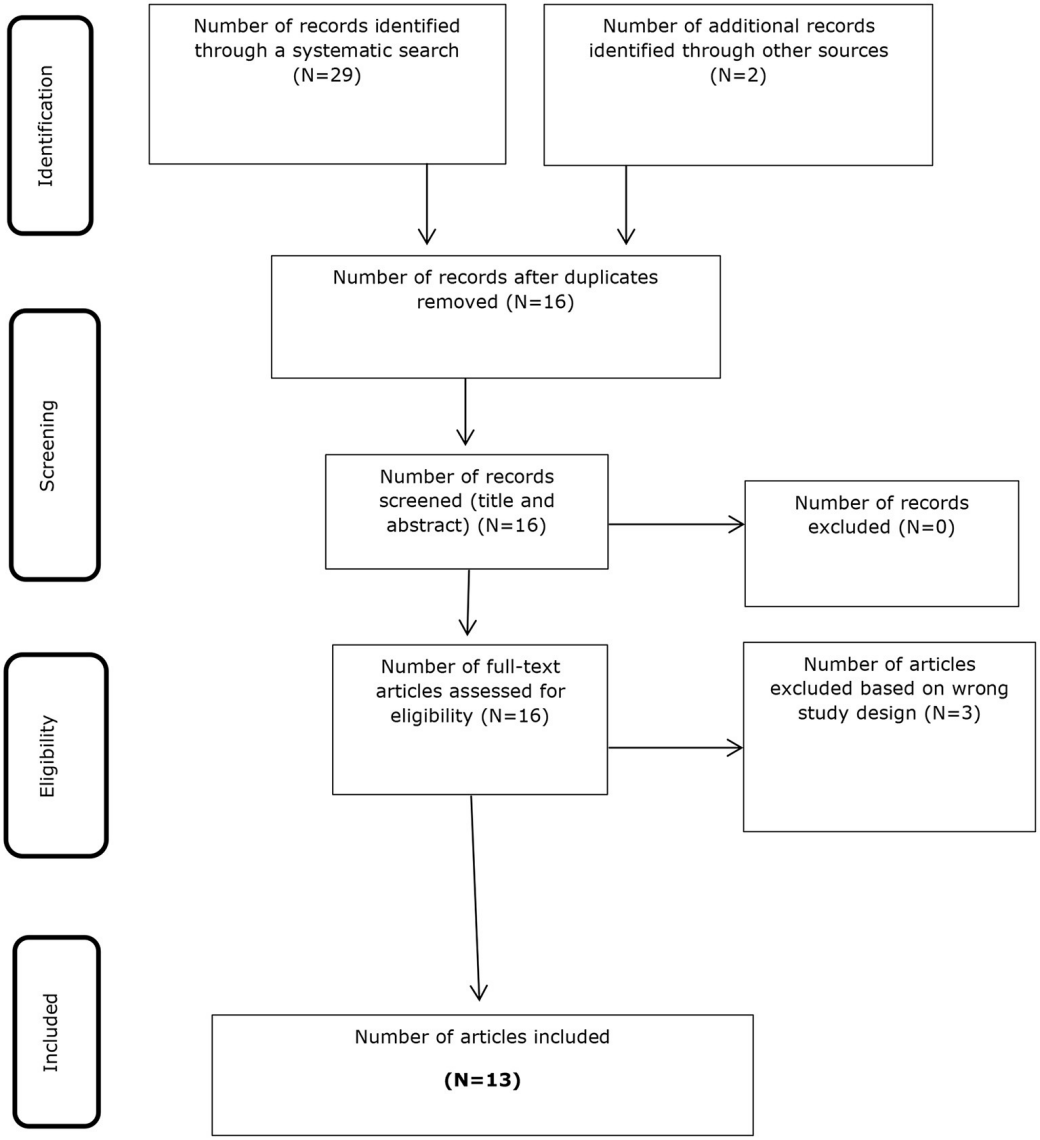
PRISMA study flow diagram.

The literature search yielded 29 articles, of which 13 met the inclusion criteria and were included in the review, which is summarized in the PRISMA flow chart (Figure 1), with an overview of the characteristics and outcomes of the included studies provided in Table 1. Five studies investigated the effects of BFRT on tendon pathologies, three on patellar tendinopathy, including one case series (Skovlund et al., 2020) and two case reports (Sata, 2005; Cuddeford and Brumitt, 2020). Two case reports investigated BFRT with tendon ruptures, one on biceps tendon rupture (Wentzell, 2018) and one on Achilles tendon rupture (Yow et al., 2018). Eight studies investigated the effects of BFRT on healthy tendons, five on the Achilles tendon, including four RCTs (Centner et al., 2019b; Chulvi-Medrano et al., 2020; Gavanda et al., 2020; Picon-Martinez et al., 2021) and one cross-sectional study (Canfer et al., 2021), one RCT on the patellar tendon (Centner et al., 2021), one RCT on the supraspinatus tendon (Brumitt et al., 2020), and one cohort study on the patellar tendon (Kubo et al., 2006). The sample sizes of included studies ranged from 1 to 56, with only 12 participants in total for tendon pathologies out of a total of 292 participants, with most included participants having healthy tendons. All included studies investigated the effects of a LL-BFRT intervention, five in isolation (Sata, 2005; Wentzell, 2018; Yow et al., 2018; Cuddeford and Brumitt, 2020; Skovlund et al., 2020) four compared with LL-RT (Brumitt et al., 2020; Chulvi-Medrano et al., 2020; Gavanda et al., 2020; Canfer et al., 2021), three compared with HL-RT (Kubo et al., 2006; Centner et al., 2019b, 2021), and one with both LL-RT and HL-RT (Picon-Martinez et al., 2021). The duration of BFRT interventions ranged from a single session to 14 weeks. The most common exercises used for the BFRT interventions were, plantarflexion calf raises (8), leg press (4), and knee extension (2).

### Outcome Measures

Four studies assessed pain as an outcome measure with BFRT, two with VAS scales and two with NRS-P scales. Patient reported function scales were assessed in three studies, with two using the Victorian Institute of Sport Assessment Patellar (VISA-P) for patellar tendinopathy and one using both the Disabilities of the Arm, Shoulder, and Hand (DASH) and Mayo Elbow Performance Index score for biceps tendon rupture. Seven studies assessed strength as an outcome, with five using dynamometry, one using 1-RM testing and one using an isokinetic Biodex system. Eight studies used ultrasound (US) to assess tendon mechanical and morphological properties, with tendon thickness the most assessed tendon outcome, measured in five studies, with four studies also assessing tendon stiffness. Muscle properties were assessed in four studies, with three studies using US to assess muscle volume or cross-sectional area and one using magnetic resonance imaging (MRI). One study used thermograms to assess Achilles tendon skin temperature. One study assessed power using an isokinetic Biodex system. One study used MRI to assess tendon signal intensity (echogenicity).

### Outcomes

The five studies that investigated the effects of a BFRT intervention on a tendon pathology, all found clinical improvements in pain, function, and muscle strength for included patients, with athletic patients being able to return to sport. The eight studies that investigated BFRT on populations with healthy tendons, all found beneficial physiological effects on tendon morphology and mechanical properties, including beneficial changes in tendon stiffness, thickness, vascularity, signal intensity, and skin temperature. However, two studies did not find changes in tendon stiffness following BFRT. Several studies also found increases in muscle volume and cross-sectional area which was associated with increases in muscular strength and decreased pain levels.

### Training Parameters

All included studies applied a BFRT cuff to either the proximal or distal limb of the targeted joint, however there were wide variances in the type and size of cuffs used, with cuff width ranging from 7 to 15 cm. Occlusion pressure was calculated as either absolute pressure ranging from 80 to 180 mm Hg, or a percentage of arterial occlusion ranging from 30 to 80%. There were wide variances in the sets and repetitions of prescribed exercises, with the commonly recommended BFRT protocol of four sets of 30, 15, 15, and 15 repetitions being implemented in seven studies. The number of sets across studies ranged from 3-6, with repetitions ranging from 5 to 30, with one study using muscular failure instead of predefined repetitions. Training frequency ranged from 2 to 7 times per week, with training intensity most commonly at 30% of 1-RM, as applied in nine studies. Most studies did not report how the training stimulus was progressed, with two studies progressively increasing occlusion pressure, one increasing percentage of 1-RM (20–35%), and two studies reported using small increases in external weight. Rest time between exercises was 30 s in seven studies and 1 min in two studies, with four studies reporting 3 min rest between different exercises, with three of these studies deflating cuff pressure between exercises.

## Discussion

The main findings of this scoping review were that despite the dearth of studies available on the effects of BFRT on tendons, studies do indicate that BFRT can produce beneficial effects on tendons. Preliminary evidence from case series and case reports indicates that BFRT may be helpful for improving clinical outcomes such as pain in function in rehabilitation of tendinopathy and tendon ruptures, however no RCTs have been conducted in these populations. The evidence for beneficial changes in healthy tendons is more robust due to several RCTs on the topic, showing beneficial physiological effects on tendon morphology and mechanical properties, including increases in tendon stiffness, with reductions in tendon thickness, vascularity, signal intensity (echogenicity) and skin temperature. Although it is unclear if these beneficial effects found in healthy tendons would also occur with pathological tendons, the preliminary evidence suggesting clinical improvement with BFRT in tendon pathology, is suggestive of potential comparable physiological benefits in tendon pathology. There is a clear need for further interventional studies of BFRT in tendinopathy and tendon rupture rehabilitation, with high quality large scale RCTs required to reach definitive conclusions and recommendations for BFRT in tendon pathology. However, there is a clear scientific rationale for the potential of clinical improvements in tendon pathology with BFRT as evidenced by the beneficial effects seen in healthy tendons, and the improvement of clinical outcomes with BFRT in other musculoskeletal disorders (Ohta et al., 2003; Bryk et al., 2016; Giles et al., 2017; Ferraz et al., 2018; Korakakis et al., 2018a; Ferlito et al., 2020; Grantham et al., 2021). Given the increased research interest and clinical use of BFRT in musculoskeletal rehabilitation for non-tendon pathologies, the dearth of available studies applying BFRT to tendon pathologies could be considered somewhat surprising. This is particularly relevant considering resistance training is considered the gold-standard first-line treatment intervention for tendinopathies, particularly Achilles and patellar tendinopathy, due to a plethora of evidence showing the clinical efficacy of resistance training such as eccentric and heavy slow resistance training (Burton and McCormack, 2021). Perhaps the belief that resistance training in tendinopathy must include high training loads has been a limiting factor in the application of LL-BFRT and could explain why it is an underutilized tool in tendon rehabilitation.

The evidence from RCTs comparing LL-BFRT with HL-RT, suggests comparable outcomes for improving muscle and tendon properties (Centner et al., 2019b, 2021), with these changes possibly serving as the mechanisms to explain the clinical benefit seen with BFRT in the case reports in tendinopathy and tendon rupture rehabilitation. The first RCT investigating the effects of LL-BFRT compared to HL-RT in patellar tendinopathy has been registered in Denmark, by the authors who conducted the positive case series included in this review (Skovlund et al., 2020). This trial will be the first step in determining if a shift is required in the tendinopathy rehabilitation field, from the belief that HL-RT is a prerequisite for improving outcomes in tendinopathy, to a possible future where both HL-RT and LL-BFRT are both viable rehabilitation methods, giving clinicians and patients more options and choice during rehabilitation. This may be particularly relevant for non-athletic patients who are unaccustomed to training with heavy loads, sedentary elderly patients, or those who may have contraindications to heavy training and those with an acute painful or reactive tendinopathy or recent tendon rupture, who would be unable to tolerate the loads required with HL-RT. In the rehabilitation of ACL ruptures, LL-BFRT has been found to be a beneficial training method for increasing muscular adaptations in those who have difficultly performing HL-RT (Palmieri-Smith and Lepley, 2015). Furthermore, LL-BFRT has been shown to attenuate pain, increase strength and improve function in rehabilitation for hospital inpatients (Ladlow et al., 2018), ACL rupture (Patterson et al., 2019), patellofemoral pain (Constantinou et al., 2022), rheumatoid arthritis (Rodrigues et al., 2020), ankle fractures (Larsen et al., 2021), and knee osteoarthritis (Ferraz et al., 2018), suggesting pain improvement may be possible with lower training loads in tendon injuries without requiring all patients to undertake HL-RT.

Included studies used low training intensities, with most programming training based on a percentage of an individual's 1-RM, typically 30%, which is congruent with loads between 20 and 40% of 1RM which are typically recommended in the BFRT literature (Kilgas et al., 2019). It is well-established that LL-BFRT requires a higher volume of repetitions to derive physiological adaptations (Kraemer and Ratamess, 2004), with the 30-15-15-15 program of 75 repetitions per set, completed with four sets typically recommended (Patterson et al., 2019). Whilst seven studies implemented this regime, the number of sets across studies ranged from 3 to 6, with repetitions ranging from 5 to 30, with one study using muscular failure instead of predefined repetitions. It is unclear if training to volitional muscular failure with BFRT is required to derive adaptations, with previous BFRT studies suggesting it may be unnecessary (Patterson et al., 2019). Previous studies have shown that muscular failure is not required for muscle hypertrophy, with overall training load volume considered more relevant for augmenting hypertrophy (Schoenfeld et al., 2017, 2019; Lasevicius et al., 2018, 2019). Details on rest periods and whether cuff pressure was maintained or deflated between sets and exercises varied across studies. However, previous research has shown that rest with an inflated or deflated cuff are viable options (Yasuda et al., 2013), although longer rest periods may reduce metabolic stress and therefore limit potential adaptations compared to short rest periods (Loenneke et al., 2010a,b; Patterson et al., 2019). Despite large variances in the BFRT arterial occlusion pressure of included studies which ranged from 30 to 80%, recommendations for occlusion pressure in the literature do range from 40 to 80% (Loenneke et al., 2011; Patterson et al., 2019), suggesting pressure should be individualized based on measures of arterial pressure and comfort levels (Jessee et al., 2016; Mattocks et al., 2017).

This review has several limitations, particularly the small number of studies included, with only five studies on tendon pathology, all being case series or case reports, highlighting the need for future high-quality studies with larger sample sizes, as there are no RCTs on BFRT in tendon pathology currently available. Future studies should also investigate the effects on specific subgroups known to be at increased risk for tendon injuries such as athletes. There was considerable heterogeneity of the BFRT parameters implemented in studies, with standardized methods and reporting of interventions required in future BFRT studies in tendon rehabilitation to enhance clinical translation of the research interventions. The longest follow-up times of included BFRT interventions were 14 weeks, with much longer follow up times required to assess the long-term adaptations and outcomes of BFRT on healthy and pathological tendons. Methods for monitoring and recording adherence to BFRT should also be emphasized in future studies as several included studies did not report the adherence level to BFRT, which may vary due to perceptual responses and comfort which may affect reported clinical outcomes (Loenneke et al., 2011; Martin-Hernandez et al., 2017; Freitas et al., 2021b; Suga et al., 2021).

## Perspectives

The superiority of LL-BFRT over standard LL-RT for muscular adaptations have been previously highlighted (Takarada et al., 2002; Madarame et al., 2008; Abe et al., 2010a,b; Yasuda et al., 2010; Centner et al., 2019c; Lambert et al., 2021), with findings from this review suggesting the same may be true for tendon adaptations. However, it remains unclear whether LL-BFRT or standard HL-RT is a superior method for inducing muscular adaptations, with some studies finding equal benefit for muscle strength gains (Lixandrao et al., 2015; Vechin et al., 2015; Curran et al., 2020; Gronfeldt et al., 2020; Hill et al., 2020) and others suggesting HL-RT is a superior method (Hughes et al., 2019b). Some studies included in this review suggest that the tendon adaptations in the healthy Achilles and patellar tendon following LL-BFRT are comparable to those evoked by HL-RT, which is an encouraging finding for the field of tendon rehabilitation (Centner et al., 2019b, 2021). However, these comparable beneficial tendon adaptations found in the high-quality RCTs on healthy tendons need to be investigated in high-quality RCTs in tendon pathology before conclusions can be drawn and recommendations made. Such findings, if found to be comparable and translate in tendon pathology may require a paradigm shift in the tendinopathy rehabilitation field in relation to the prescription of resistance training interventions, particularly for select populations not able to tolerate the standard and proven HL-RT interventions (Loenneke et al., 2013).

## Conclusion

Despite a dearth of studies to date on the effects of BFRT on healthy tendons and in tendon pathologies such as tendinopathy, preliminary evidence for beneficial effects of BFRT on tendons and clinical outcomes is encouraging. As BFRT is a relatively novel method, particularly its application in musculoskeletal rehabilitation, definitive conclusions, and recommendations on BFRT in tendon rehabilitation cannot be made at present, which should be addressed in future research, due to the potential therapeutic benefits highlighted in this review. The addition of LL-BFRT as a viable rehabilitation method in tendinopathy rehabilitation would be complimentary to currently utilized HL-RT interventions and provide more rehabilitation options for patients unable to tolerate HL-RT during tendon rehabilitation.

## Author Contributions

IB conceptualized the work, developed the methods, search strategy, and framework for the review. IB and AM contributed to the development of the research questions and the study design. Both authors developed the first and subsequent drafts of the manuscript and reviewed and approved the manuscript.

## Conflict of Interest

The authors declare that the research was conducted in the absence of any commercial or financial relationships that could be construed as a potential conflict of interest.

## Publisher's Note

All claims expressed in this article are solely those of the authors and do not necessarily represent those of their affiliated organizations, or those of the publisher, the editors and the reviewers. Any product that may be evaluated in this article, or claim that may be made by its manufacturer, is not guaranteed or endorsed by the publisher.
