# Supplementary material for: Blood Flow Restriction Resistance Training in Tendon Rehabilitation: A Scoping Review on Intervention Parameters, Physiological Effects, and Outcomes
Source: Front Sports Act Living. 2022 Apr 25;4:879860. doi: 10.3389/fspor.2022.879860 (PMC9083008; doi:10.3389/fspor.2022.879860)
Supplement: Supplementary file 1 [file Table_1.pdf]

Appendix 1: Search strategy

- Information sources and search strategy.

Databases: MEDLINE (PubMed), CINAHL, AMED, EMBase, SPORTDiscus CENTRAL of Cochrane Library

Search fields: Title, abstract, key words

Search terms (database subject headings)

1. - “Blood Flow Restriction” OR “Kaatsu” OR “Occlusion Training” 2 - “tendon” OR “tendinopathy” OR “tendon rupture”

3. 1 AND 2
